# Resistance of strongylid nematodes to anthelmintic drugs and driving factors at Czech goat farms

**DOI:** 10.1186/s12917-021-02819-8

**Published:** 2021-03-05

**Authors:** Jaroslav Vadlejch, Iveta Angela Kyriánová, Marián Várady, Johannes Charlier

**Affiliations:** 1 Department of Zoology and Fisheries, Faculty of Agrobiology, Food and Natural Resources, Czech University of Life Sciences Prague, Kamýcká 129, 165 00 Prague, Suchdol Czech Republic; 2 Institute of Parasitology, Slovak Academy of Sciences, 040 01 Košice, Slovak Republic; 3 Kreavet, Hendrik Mertensstraat 17, 9150 Kruibeke, Belgium

**Keywords:** Caprine gastrointestinal nematodes, Control, Dual resistance, In vitro test, Stocking rate, Farmer experience

## Abstract

**Background:**

Strongylid nematode infections may negatively affect both animal health and welfare, with deleterious consequences for livestock productivity. Many farmers in recent decades have relied on anthelmintics as the sole strategy of control, but the intensive use of these chemotherapeutics has led to the development of anthelmintic resistance (AR). Knowledge of both the efficacy of anthelmintics and factors promoting AR are essential to effectively control nematode infections, but no information on these topics for goats in the Czech Republic (CR) is available. This survey aimed to determine the occurrence of AR at Czech goat farms and to identify risk factors for the development of AR. A total of 24 herds of dairy goats across the CR were evaluated using in vitro tests for detecting AR, and a questionnaire survey was carried out to evaluate factors associated with AR.

**Results:**

Resistance against benzimidazoles was confirmed at 18 (75%) farms, and the level of resistance was high in four (22%) of the affected herds based on the egg hatch test. Ivermectin-resistant nematodes were detected in 13 (54%) herds using the larval development test; *Teladorsagia*/*Trichostrongylus* and *Haemonchus* were the predominant types of resistant larvae. Eight (62%) of the affected herds were evaluated as highly resistant to ivermectin. Eleven (46%) of the herds were resistant to both benzimidazoles and ivermectin. This report is the first on dual AR in the CR. A univariate logistic regression analysis indicated that a high stocking rate and farmer inexperience were significantly associated with ivermectin and benzimidazole resistance, respectively.

**Conclusions:**

The results of our survey suggest that AR is widespread amongst herds of dairy goats in the CR, likely due to inappropriate practices of pasture and health management. AR may be an issue for expanding dairy-goat production in the CR in the near future unless both veterinary practitioners and farmers widely adopt strategies to prevent the development of AR.

**Supplementary Information:**

The online version contains supplementary material available at 10.1186/s12917-021-02819-8.

## Background

Resistance to anthelmintic drugs in gastrointestinal (GI) nematodes of farmed ruminants is widespread and is an important factor limiting ruminant production worldwide [1, 2]. Anthelmintic resistance (AR) is a large problem, particularly in the Southern Hemisphere where resistance to all major broad-spectrum anthelmintics (including triple resistance) in pastured ruminants is frequently reported [3]. Sheep production is no longer profitable at some South African and Australian farms due to AR, and farmers have had to change their livelihood strategies [3, 4]. The situation at European farms may be less pronounced, but AR in the main GI nematodes of ruminants has been recorded for all classes of anthelmintics throughout Europe [5].

AR is of particular interest in small ruminants, where it has become very common at the vast majority of sheep farms in Europe [6, 7]. Goats are more susceptible than sheep to infections with GI nematodes and are at a higher risk of AR development, because goats metabolise drugs faster and the spectrum of drugs licensed for this ruminant livestock species is limited [8, 9]. AR, however, has been less documented in goat herds than sheep flocks. Local reports or nationwide surveys for cases of resistance in goat GI nematodes to benzimidazoles (BZs), macrocyclic lactones (MLs), and levamisole (LEV) have been reported from the majority of European countries [10], but the prevalence and level of AR vary amongst the countries. Resistance in goat GI nematodes to BZs is widespread (≥80% prevalence) in Denmark [11] and Slovakia [12]. BZ resistance has become a serious problem primarily in France; all goat herds investigated were affected in some regions [13, 14], and the problem was not less pronounced in farms under extensive management [15]. The frequency of BZ resistance in goats in other countries such as Norway has been determined to be low [16]. Resistance against MLs has mainly been described in Switzerland and Denmark. None of the 43 goat herds examined by Murri et al. [17] in Switzerland were susceptible to eprinomectin, and the mean reduction in faecal egg count after treatment was only 40%. Artho et al. [18] confirmed resistance to doramectin in 29% of the Swiss goat herds examined and suspected AR in another 33% of the farms surveyed. Holm et al. [11] reported both a high (80%) prevalence and level of ivermectin (IVM) resistance in Denmark. In contrast, full efficacy of MLs against goat GI nematodes was recorded in France [19] and Norway [16]. Resistance against two classes of anthelmintics has been reported at goat farms in Denmark [11, 20] and France [15], and triple resistance was recorded at one Slovak goat farm with animals imported from New Zealand [21].

Alternative approaches to control GI nematode infections in grazing livestock are available [22, 23], but anthelmintic treatment still remains the most effective. The emergence of resistance may be inevitable at any farm applying chemotherapeutics, so identifying and understanding the factors associated with AR development and the preservation of anthelmintic efficacy are urgently needed. Such factors have been thoroughly investigated in sheep [24], and guidelines for the sustainable control of nematodes have been developed based on this information, e.g. SCOPS (Sustainable Control of Parasites in Sheep) [25]. In contrast, risk factors for the development of AR at goat farms under European conditions have not yet been fully determined. AR is regionally dependent due to the large differences in environment/climate, management practices, animal breeds, and production between ruminant farms within Europe [16, 26], so evaluating risk factors at the regional or at least national level is essential.

Data for AR in sheep within the Czech Republic (CR) are limited, and no information about this topic is available for goats. Chroust [27] recorded the first case of both BZ and LEV resistance in sheep in the CR. AR to BZ and LEV was then detected at Czech sheep farms at the beginning of this century [28] and later to BZs [29]. Vadlejch et al. [30] conducted the most recent survey at Czech sheep farms using a combination of in vivo and in vitro tests for detecting BZ and IVM resistance.

We conducted a survey to gather information on both anthelmintic efficacy and risk factors for developing AR at Czech goat farms where the interest in goat farming is steadily increasing and the demand for goat milk and other dairy products are growing. Our objectives were to: i) evaluate the efficacy of BZs and IVM at dairy-goat farms using in vitro tests, and ii) identify associations between AR and farmer, management, and control factors at the surveyed farms.

## Results

### In vitro tests

Both in vitro tests for detecting AR were possible at all goat farms due to sufficient numbers of nematode eggs recovered from the faecal material. The egg hatch test (EHT) was performed in triplicate, but the larval development test (LDT) was only performed in duplicate for all drug concentrations due to a lower number of nematode eggs isolated. The mean proportion of eggs hatching in the control wells of all tissue-culture plates (EHT) was 98 ± 2%, and the mean proportion of infective larvae in the control wells of all microplates (LDT) was 79 ± 8%.

Resistance to BZ was detected in 75% (18 of 24) of the herds using the EHT. The medium effective dose (ED_50_) of thiabendazole (TBZ) ranged from 0.01 to 0.62 μg.ml^− 1^ (Table 1), and the mean ED_50_ in the BZ-resistant herds was 0.34 μg.ml^− 1^. A maximum of 7% of BZ-susceptible eggs hatched in the plates containing 0.3 μg TBZ.ml^− 1^, whereas 21–74% of BZ-resistant eggs hatched at this concentration. The majority (78%) of the BZ-resistant herds had a moderate level of resistance based on the hatching categories (see Data analysis and assessment of AR and risk factors), and the other herds (22%) were evaluated as highly resistant. IVM resistance was detected in 54% (13 of 24) of the herds based on the selected threshold. Medium lethal dose (LD_50_) ranged from 0.18 to 184.2 ng.ml^− 1^ (Table 2), and mean LD_50_ in the IVM-resistant herds was 99.2 ng.ml^− 1^. Sixty-two percent of the IVM-resistant herds had a high level of resistance (LD_50_ ≥ 81.9 ng.ml^− 1^ in our survey, see Data analysis and assessment of AR and risk factors). Infective larvae were recovered from all microplate wells containing 21.6 ng IVM-AG.ml^− 1^, which were considered to be resistant (Table 2). *Teladorsagia*/*Trichostrongylus* were the predominant type of resistant larvae in the affected herds, even though the number of infective larvae (L_3_) varied considerably and a mixture of nematode species was detected in most wells containing 21.6 ng ivermectin aglycone (IVM-AG).ml^− 1^ (Table 2). *Oesophagostomum*/*Chabertia* larvae in five resistant herds were identified in the wells containing 21.6 ng IVM-AG.ml^− 1^, but low percentages of these larvae were recovered. Resistance to both BZ and IVM was detected at 46% (11 of 24) of the goat farms using the in vitro tests. A high level of resistance to both drug classes was identified in two (18%) of all dual-resistant herds.

**Table 1:** Assessment of benzimidazole resistance at the dairy goat farms in the Czech Republic using the egg hatch test

Farm	ED_50_ ± SD	ED_99_ ± SD	Egg hatching ± SD (%) ^a^		Resistance status
	(μg.ml^-1^)	(μg.ml^-1^)	0.1 μg.ml^-1^	0.3 μg.ml^-1^	
1	0.35 ± 0.05	11.72 ± 2.09	52.9 ± 5.4	37.0 ± 6.1	R
2	0.59 ± 0.07	5.69 ± 0.49	91.8 ± 1.2	73.5 ± 8.9	R
3	0.24 ± 0.03	2.19 ± 0.64	35.3 ± 1.5	21.1 ± 4.1	R
4	0.37 ± 0.07	14.19 ± 1.57	78.5 ± 1.5	53.2 ± 4.9	R
5	0.35 ± 0.05	1.00 ± 0.37	62.6 ± 13.0	48.8 ± 2.6	R
6	0.01 ± 0.01	0.36 ± 0.04	0.3 ± 0.2	0.0 ± 0.0	S
7	0.01 ± 0.02	0.03 ± 0.07	0.8 ± 0.3	0.0 ± 0.0	S
8	0.26 ± 0.03	0.56 ± 0.04	80.3 ± 6.9	24.4 ± 0.0	R
9	0.30 ± 0.01	19.24 ± 3.66	29.7 ± 6.6	28.1 ± 4.3	R
10	0.28 ± 0.04	2.07 ± 0.60	72.2 ± 6.7	42.0 ± 8.9	R
11	0.30 ± 0.03	2.15 ± 0.58	56.4 ± 3.8	28.8 ± 0.4	R
12	0.28 ± 0.03	6.79 ± 0.56	68.9 ± 3.6	40.3 ± 4.1	R
13	0.25 ± 0.05	1.68 ± 0.11	62.6 ± 6.5	32.5 ± 2.9	R
14	0.36 ± 0.06	17.52 ± 3.61	69.7 ± 7.5	53.5 ± 6.2	R
15	0.01 ± 0.01	0.03 ± 0.01	5.0 ± 0.8	2.1 ± 1.7	S
16	0.39 ± 0.08	0.94 ± 0.07	51.2 ± 4.0	43.4 ± 10.1	R
17	0.62 ± 0.05	14.10 ± 1.15	81.4 ± 3.6	69.1 ± 1.7	R
18	0.39 ± 0.06	1.02 ± 0.17	79.2 ± 3.2	48.6 ± 9.3	R
19	0.01 ± 0.01	0.04 ± 0.01	12.1 ± 3.4	7.1 ± 4.8	S
20	0.37 ± 0.07	5.19 ± 0.94	43.3 ± 0.7	27.8 ± 5.5	R
21	0.25 ± 0.05	2.70 ± 0.59	55.4 ± 4.3	23.6 ± 2.8	R
22	0.01 ± 0.00	0.57 ± 0.18	0.4 ± 0.1	0.0 ± 0.0	S
23	0.23 ± 0.04	15.86 ± 1.63	56.2 ± 5.4	45.7 ± 2.2	R
24	0.01 ± 0.03	0.03 ± 0.02	11.8 ± 2.6	7.3 ± 2.7	S

Effective doses (ED_50_ and ED_99_) of thiabendazole are expressed as the mean of three measurements ± the standard deviation (SD), ^a^ percentage of nematode eggs that hatched at thiabendazole concentrations of 0.1 and 0.3 μg.ml^-1,^ *S* herd susceptible to benzimidazoles, *R* herd resistant to benzimidazoles (ED_50_ > 0.2 μg/ml TBZ and > 20% of the eggs hatched at 0.3 μg.ml^-1^)

**Table 2:** Assessment of ivermectin resistance at the dairy goat farms in the Czech Republic using the larval development test

Farm	LD_50_ ± SD	LD_99_ ± SD	Larval development ± SD (%) ^a^	Resistance status	Recovered nematode species ^b^
	(ng.ml^-1^)	(ng.ml^-1^)			
1	0.22 ± 0.03	1.62 ± 0.28	0.0 ± 0.0	S	–
2	0.34 ± 0.08	5.80 ± 0.64	0.0 ± 0.0	S	–
3	33.20 ± 6.22	98.41 ± 6.01	4.5 ± 0.4	R	*Tel*/*Tri*
4	2.21 ± 0.21	12.54 ± 2.69	0.0 ± 0.0	S	–
5	41.80 ± 1.34	101.39 ± 11.46	17.0 ± 2.7	R	*Tel*/*Tri*, *Hae*
6	180.10 ± 35.14	226.00 ± 6.77	79.3 ± 5.1	R	*Hae*, *Tel*/*Tri*, *Oe*/*Cha*
7	10.02 ± 1.28	76.31 ± 4.43	11.8 ± 1.9	R	*Tel*/*Tri*, *Hae*, *Oe*/*Cha*
8	11.24 ± 0.66	52.18 ± 4.74	17.0 ± 2.8	R	*Tel*/*Tri*, *Hae*, *Oe*/*Cha*
9	184.20 ± 22.45	266.44 ± 74.24	80.7 ± 6.3	R	*Tel*/*Tri*, *Hae*
10	132.20 ± 14.42	604.80 ± 164.47	57.5 ± 2.2	R	*Tel*/*Tri*, *Hae*
11	81.90 ± 34.72	436.59 ± 24.25	68.4 ± 1.7	R	*Tel*/*Tri*, *Hae*, *Oe*/*Cha*
12	156.30 ± 7.21	258.00 ± 18.13	34.1 ± 3.6	R	*Tel*/*Tri*, *Hae*
13	153.50 ± 21.85	549.92 ± 8.03	44.8 ± 5.9	R	*Tel*/*Tri*, *Hae*
14	178.10 ± 14.78	228.20 ± 4.91	40.0 ± 4.3	R	*Hae*
15	0.30 ± 0.12	2.19 ± 0.23	0.0 ± 0.0	S	–
16	0.18 ± 0.03	0.31 ± 0.06	0.0 ± 0.0	S	–
17	107.10 ± 9.97	352.80 ± 64.59	72.4 ± 6.8	R	*Tel*/*Tri*, *Hae*, *Oe*/*Cha*
18	4.27 ± 0.01	16.77 ± 2.72	0.0 ± 0.0	S	–
19	3.77 ± 0.06	40.23 ± 1.20	0.0 ± 0.0	S	–
20	2.19 ± 0.23	5.70 ± 0.20	0.0 ± 0.0	S	–
21	20.10 ± 3.18	249.90 ± 13.19	3.5 ± 1.4	R	*Tel*/*Tri*
22	2.16 ± 0.20	7.60 ± 0.45	0.0 ± 0.0	S	–
23	2.12 ± 0.19	11.60 ± 1.01	0.0 ± 0.0	S	–
24	2.74 ± 0.30	5.28 ± 0.65	0.0 ± 0.0	S	–

Lethal doses (LD_50_ and LD_99_) of the ivermectin aglycone expressed as the mean of two measurements ± the standard deviation (SD), ^a^ total percentage of infective larvae detected at an ivermectin aglycone concentration of 21.6 ng.ml^-1^, *S* herd susceptible to ivermectin, *R* herd resistant to ivermectin (LD_50_ > 21.6 ng.ml^- 1^ and > 30% of larvae developed to L_3_ stage at 21.6 ng.ml^- 1^), ^b^ species richness of infective larvae recovered at an ivermectin aglycone concentration of 21.6 ng.ml^-1^; Tel/Tri *Teladorsagia*/*Trichostrongylus,* Hae *Haemonchus,* Oe/Cha *Oesophagostomum*/*Chabertia,* the larvae are sorted by their percent occurrence in the sample

### Questionnaire survey and assessment of risk factors

The putative farmer, management, and control factors associated with AR are summarised in Table 3. The herds represented all regions of the CR and the majority of environmental conditions typical for this country, including lowlands and mountainous areas. Altitudes of the farms ranged from 200 to 840 m above sea level. Herd size ranged from 20 to 980 goats, with a mean of 102 does. The farms comprised both conventional and organic farming systems, partitioned equally. The goats were grazed at all farms, and pastures had a mean area of 14.1 ha (range 0.8–90 ha). Sharing paddocks with other ruminants (solely sheep) was common at only five (21%) farms, but contact with wild ruminants could not be excluded for any of the herds. The farms differed considerably in the number of goats on a paddock (1–62 goats/ha), and the mean stocking rate was 13 goats per hectare. This factor of pasture management was significantly associated with IVM resistance (OR [95% CI] = 1.09 [1.00–1.24], *P* = 0.04). Pasture rotation was regularly practised at half of the farms, and animals were not drenched at any farm when moved to a new paddock. Kidding occurred mainly from January to March, and goats were turned out on pasture in April or May. Animals were grazed from spring to autumn (October or November) at 17 (71%) farms, and at the remaining herds had year-round access to pasture, depending on the meteorological conditions. All farms had a closed herd turnover, but a new buck was briefly introduced to the herd for mating at most (90%) of the farms.

**Table 3:** Frequency distribution of putative risk factors associated with anthelmintic resistance, and prevalence of resistance to anthelmintic drugs at the surveyed farms

Factor	Farm distribution (%)	Anthelmintic resistance at farms (%)^a^		
		Benzimidazole	Ivermectin	Dual
Type of production		*P* = 0.34	*P* = 1	*P* = 1
Conventional	58	64	57	43
Organic	42	90	50	50
Stocking rate		*P* = 0.64	*P* = 0.04	*P* = 1
≤ 8 goats per hectare	50	67	42	42
≥ 9 goats per hectare	50	83	67	50
Pasture rotation		*P* = 0.64	*P* = 0.41	*P* = 1
Yes	50	67	67	50
No	50	83	42	42
Farmers’ educational level		*P* = 1	*P* = 0.39	*P* = 0.66
≤ Middle school	71	76	74	35
University	29	71	71	57
Farming Experience		*P* = 0.04	*P* = 0.64	*P* = 0.31
≤ 5 years	16	100	75	75
6–10 years	42	70	60	50
> 10 years	42	70	40	30
Faecal examination		*P* = 1	*P* = 0.41	*P* = 0.70
Yes	58	71	64	50
No	42	80	40	40
Regular deworming		*P* = 1	*P* = 1	*P* = 1
Yes	83	75	50	50
As required	13	100	33	33
No	4	0	0	0
Deworming frequency		*P* = 1	*P* = 1	*P* = 1
Once a year	25	100	50	50
Twice a year	50	58	50	50
Other frequency	25	67	33	33
Targeted selective treatment		*P* = 0.54	*P* = 1	*P* = 1
Yes	17	100	50	50
No	83	61	48	39
Animals weighed before drug application		*P* = 1	*P* = 0.18	*P* = 0.48
Yes	9	100	0	0
No	91	70	57	48
Drug dose		*P* = 0.12	*P* = 0.38	*P* = 0.09
Sheep dose	35	100	75	75
Sheep dose × 2	65	67	47	33

*P* (probability) values, significant association *P* ≤ 0.05, ^a^ AR status referred to Tables 1 and 2

The age of farmers ranged between 28 and 65 years, with a mean of 45 years. The majority (71%) of farmers had completed elementary or secondary education, and only eight farmers (33%) were educated in agricultural fields. Experience in dairy-goat farming varied highly from three to 46 years; the farmers had reared animals for an average of 9.5 years. Our results indicated a significant negative association between farming experience and BZ resistance (OR [95% CI] = 0.89 [0.76–1.00], *P* = 0.04).

Anthelmintics were applied twice a year at twelve (50%) farms. All these farms had a predetermined treatment plan; the vast majority (92%) drenched herds in spring when turned out on pasture and thereafter in autumn at housing. Six herds (25%) were treated once annually, and drenching in these herds was scheduled either in spring (33% of herds) or winter during the housing period (67% of herds). The remaining herds (25%) were not drenched regularly every year, and no anthelmintic had been applied for the last five years at one farm (4%). Farmers relied mainly on BZs (83% of farms) and MLs (70% of farms). Nearly all farms treated goats twice annually, rotating between BZs and MLs; oral drenches of BZs were applied by farmers in spring, and injectable IVM was administered by a veterinary practitioner in autumn or winter. The other anthelmintic class (LEV) was used in only four (17%) herds. Unlike other EU countries where the use of injectable IVM is prohibited in dairy animals, this route of IVM administration is allowed in animals during the drying period in the CR. Animal live weight for calculating drug dose was estimated visually at most (91%) of the farms, and the standard dose (so-called sheep dose) of the drug was applied at eight (35%) farms following the manufacturers’ recommendations. More farms had AR in the group receiving the sheep dose (vs. sheep dose × 2), but these associations were not significant (*P* = 0.12 and 0.09 for BZ and dual resistance, respectively). Only four (17%) farmers declared that treatments were used strictly selectively. This factor was not significantly associated with resistance to BZs (*P* = 0.14) or IVM (*P* = 0.77) or with dual resistance (*P* = 0.92). Fourteen (58%) farmers occasionally sent faecal samples to a service laboratory for examination, and nine of these farmers (64%) drenched animals based on the results of the examinations. Drug efficacy after anthelmintic treatment is not commonly evaluated at Czech goat farms.

## Discussion

The number of goat farms is growing globally due to the expanding demand for goat dairy products [31], and the CR is no exception. The Czech goat inventory has almost doubled since 2009 [32]. There were 28,174 goats in the CR in 2017, distributed among 8018 herds; 87% comprised only hobby farms with a maximum of 10 animals. Dairy farming has a predominant role in the CR; approximately 20% (6104 in 2017) of the goats are annually registered for monitoring animal performance. The White Shorthaired goat is the predominant (42%) breed in the monitoring of animal performance [33]. Goats are known for their sensitivity to parasitic infections. Monitoring and optimising practices of parasite control in this growing sector, including the rational use of anthelmintics to delay the emergence of resistance, are therefore timely. AR in goats has been well documented in several European countries, but data for the CR are lacking. Our survey provides a first assessment of the resistance of strongylid nematodes to benzimidazoles and ivermectins in Czech goat herds.

We identified a high prevalence of BZ resistance, and IVM and dual resistance were moderately prevalent. European nationwide surveys have found that resistance against BZs is already well established in goat herds in France [14, 15], Slovakia [12], and Denmark [11]. BZ resistance is as widespread in these countries as in our survey, mostly due to the strong reliance on BZs, e.g. 97% of French goat farmers use BZs, with this anthelmintic class representing > 84% of annual treatments [34]. Many farmers prefer these drugs because of their reasonable price and the short period of milk withdrawal. Resistance to MLs is much less pronounced in goats, likely because the vast majority of European goat farms are specialised in milk production and because the use of most MLs is limited to non-lactating animals. The prevalence of ML resistance in Switzerland is higher than the prevalence detected in our survey [17, 18] or in Denmark [11], probably associated with the preference for MLs. Eprinomectin in a pour-on formulation is especially popular in Switzerland due to the convenient route of administration and a zero period of milk withdrawal. A survey conducted by Murri et al. [17] found that 86% of Swiss farmers have used this drug in goats. Multiple AR at some European goat farms has been reported [20, 21], but the current spread of this phenomenon in European countries [35, 36] is alarming and consistent with our results.

The richness of nematode species resistant to BZs should only be evaluated using the EHT based on molecular identification of L_1_ larvae, but we did not focus on this aspect in our survey. The LDT in our survey identified multispecies resistance to IVMs; *Teladorsagia*/*Trichostrongylus* and *Haemonchus* were common at IVM-AG concentrations considered to indicate resistance. The species richness of IVM-resistant nematodes identified in this survey mirrored the nematode species identified recently in goats in the CR [37] and is consistent with the richness detected in some European goat herds resistant to MLs [20, 38]. *Haemonchus* is nevertheless a major nematode genus resistant to MLs at the majority of goat farms throughout Europe [17, 39, 40]. Multiple-resistant strains of this nematode have also been isolated from goats in several European countries [35, 36, 41]. *Haemonchus* is at a higher risk of ML resistance than other nematodes, emerging presumably due to the lower sensitivity of *Haemonchus* females to IVMs [42] and to the very high prolific potential [43] and high genetic diversity of this genus [44]. If Czech farmers continue to practise the current measures of parasite control and if climate change continues as expected, then *Haemonchus* may remain a major resistant species in the near future in goat herds in the CR.

In addition to monitoring AR, understanding the risk factors promoting the development of AR is essential in every country. The detection of these factors is unfortunately difficult, because of the multifactorial basis of AR, and requires many observations. Only a few studies have thus identified the factors contributing to the development of AR. A systematic review and meta-analysis of risk factors for AR in sheep only identified the frequency of treatment as an important factor [24]. Similar analyses have yet to be conducted for goats, but none of the putative risk factors in a survey of Swiss goat herds were associated with eprinomectin resistance [17]. Only stocking rate and farming experience in our survey were significantly associated with AR.

Overcrowding is a common predisposition of many diseases, and intensities of GI nematode infections are high when animals are assembled at high stocking rates. AR may thus be expected at high population densities of animals on pasture. Gaba et al. [45] clearly demonstrated that the higher the stocking rate, the greater the selective pressure for AR. These results are consistent with our survey. We detected a higher proportion of resistance to both anthelmintic classes evaluated (including dual resistance) when the stocking rate exceeded eight goats per hectare. This association, however, was only significant for resistance to IVM. The level of IVM resistance was five-fold higher and was present in dual nematode species in herds with high stocking rates. Our results suggest that resistance to IVM is already well established in these goat herds and that farmers should avoid the use of IVM in the future. Drug persistence could account for the significant association between stocking rate and IVM resistance in our survey. IVM is more persistent than BZs, which can accelerate the development of resistant nematode populations [46, 47]. Nematodes entering the host during the elimination of a drug are exposed to doses lower than the therapeutic dose, and high population densities of animals on a pasture may exacerbate this problem. Stocking rate should thus be an important consideration in both parasite control and the delay of AR development. Establishing general recommendations for optimal stocking rates is challenging, and farmers should take several factors into account (e.g. weight of animals, type of animal breeding, pasture size and composition, and current meteorological conditions). The frequency of resistant alleles will increase substantially at a stocking rate of 16 sheep per hectare and very high levels of AR may be expected at a stocking rate of 20 sheep [45]. Similar studies for goats have yet to be conducted, but stocking rates lower than those published for sheep may be recommended for goats due to the ability of goats to shed higher numbers of nematode eggs compared to sheep.

Many farmers in the CR (especially the young ones) have begun to raise goats in the last decade. Establishing a commercial farm requires specific knowledge and experience, which is a challenge for new farmers [48]. Acquiring sufficient practical experience in farming generally requires several years, and poor pasture and management of nutrition and health within this period may cause severe health problems in a herd. Inexperienced farmers generally consider parasites as the sole source of these problems and rely strongly on anthelmintics, so other measures to improve health (e.g. feed supplements) are not practised. Farmers frequently drench their animals when health and body conditions do not improve. Our survey identified BZ resistance at all goat farms where farmers had less than five years of experience. The relationship between these factors may be due to the preference of new farmers to use BZs instead of other drug classes. A reasonable price, a short period of milk withdrawal, and the predominant peroral formulation make BZs their drugs of choice. All low experienced farmers in our survey used these drugs almost exclusively. The probability of developing AR decreases when farmers accumulate knowledge and practical experience over time. The prevalence of BZ resistance in our study was 1.4-fold lower at farms led by experienced farmers. Similar trends were observed for IVM resistance but were not significant.

Other factors evaluated in our survey (including targeted selective treatment [TST] or underdosing) were not significantly associated with AR. TST is a promising strategy to effectively control nematode infections while maintaining anthelmintic efficacy [49, 50]. Anthelmintic treatment based on this approach is administered only to animals that are obviously suffering from nematode infections, and such treatment is beneficial to them because it enhances both their health and production [51, 52]. Our survey found evidence of BZ resistance at all farms where TST was practised and of IVM resistance at half of the farms and detected dual resistance. The choice of treatment indicator, however, is crucial for the efficacy of TST [53, 54]. Czech farmers traditionally select animals for drenching based on faecal consistency (diarrhoea), but this indicator is unreliable for TST in goats [55].

A more rapid metabolism of xenobiotics is responsible for the faster clearance of anthelmintics from goats than sheep. Drenching goats at the standard dose recommended by the manufacturer (sheep dose) will thus lead to underdosing [8, 56]. Insufficient doses of a drug permanently administered to goats is one of the most important factors responsible for the high prevalence of AR in this livestock species [9, 57, 58]. Goats should be treated at about 1.5 times the recommended sheep dose rates for MLs and at twice the recommended sheep dose rates for BZs to achieve the same therapeutic effect as in sheep [56]. Very high prevalences of resistance, including dual resistance, were detected at farms where standard sheep doses had been used for many years [14, 15, 20], but this factor was not significantly associated with AR in our survey.

## Conclusion

This first survey of AR at Czech goat farms found that AR was well established amongst strongylid nematodes. This high prevalence of AR may be a particular issue for dairy-goat production due to the limited number of anthelmintic classes licensed for goats and/or applicable to lactating animals. Anthelmintic treatment will remain the cornerstone for controlling nematode infections in the near future, so measures are required to preserve the efficacy of anthelmintics. We identified high stocking rates and farmer inexperience as two potential important factors driving the development of AR. Control programmes based on understanding parasite biology and local epidemiology of infections should be implemented to prevent the further spread of AR. Stronger knowledge transfer and capacity building in farmer and veterinary communities for sustainable methods of parasite control will be necessary. Further monitoring of AR in goats and other ruminants in the CR is recommended.

## Methods

### Farm selection and sample collection

The goat herds were selected based on known histories of parasitic burdens and on the willingness of the farm owner to participate in the survey. The vast majority of these herds had been investigated in a parasitological survey in 2017 [37]. Owners were contacted by telephone before we visited the farm to discuss specific requirements for our study. A total of 24 herds that fulfilled the following criteria were included in this survey, performed between May and October 2019: (i) goat breed (White Shorthaired), (ii) number of animals in the herd (at least 20 adults), (iii) commercial dairy production, and (iv) grazing management (access to paddock most of the day and throughout the grazing season). One organic and one conventional farm were selected from most of the 14 regions of the CR. Goats in the surveyed herds had not received any anthelmintic treatment for at least three months prior to initiation of the survey. Faecal samples were collected either directly from the rectum of the animals or from the ground immediately after an animal defecated. The samples used for detecting BZ resistance were stored strictly anaerobically to prevent the development of nematode eggs, as described by Hunt and Taylor [59], and the samples for detecting IVM resistance were transported in plastic boxes covered by lids. The faecal samples were always processed the same day they were collected. The in vitro tests were processed by only one technician to avoid biasing the results. Nematode eggs for the tests were recovered from the faeces using a double centrifugation flotation technique (850 RCF for 5 min and 780 RCF for 3 min) [60]. The eggs floating on the coverslip were removed, cleaned with deionised water, and processed as soon as possible.

### In vitro tests

#### Egg hatch test

The EHT was used for detecting BZ resistance, as recommended by the World Association for the Advancement of Veterinary Parasitology [61, 62]. The test was performed following a standardised procedure [63]. Nematode eggs were isolated in deionised water and examined under the microscope to ensure that embryonation had not yet begun. Solutions of thiabendazole (TBZ, T8904, Sigma-Aldrich) at final concentrations of 0.05, 0.1, 0.2, 0.3, and 0.5 μg.ml^− 1^ were applied to wells of 24-well tissue-culture plates containing an aqueous suspension of nematode eggs (~ 100 eggs per well). All dilutions contained 0.5% dimethyl sulphoxide (DMSO, Sigma-Aldrich). Three replicates were evaluated for each TBZ concentration, and three control wells without anthelmintic (0.5% DMSO) were included. The plates were sealed and incubated at 27 °C for 48 h. One drop (~ 10 μl) of Lugol’s solution was then added to each well both to prevent further hatching of eggs and to inactivate developed larvae. The numbers of eggs (those that failed to form larvae and those containing larvae) and L_1_ larvae in each well were determined under an inverted microscope.

### Larval development test

Resistance to IVM was assessed using the LDT. A slightly modified method from that previously described by Hubert and Kerboeuf [64] was applied. An ivermectin aglycone (IVM-AG, BIA-I1151, Tebu-bio) was used in this survey based on studies by Dolinská et al. [65, 66]. A stock solution of this drug was prepared by dissolution in DMSO. An aqueous egg suspension (~ 100 eggs per well) treated with 5 μg.ml^− 1^ amphotericin B (Sigma-Aldrich) to prevent fungal contamination, 20 μl of culture medium for larval development, and 10 μl of the IVM-AG solution at the appropriate concentration were added to each well of a 96-well microtitrer plate. A total of 12 final concentrations of IVM-AG (a two-fold serial dilution ranging from 0.084 to 173.6 ng.ml^− 1^) were investigated. The culture medium consisted of yeast extract dissolved in physiological saline solution, Earle’s solution, and lyophilised *Escherichia coli* (see 62 or 64 for details). All reagents were purchased from Sigma-Aldrich. Each sample (including the control) was tested in duplicate. The drug was replaced by 0.5% DMSO in the control wells. The plates were covered and incubated at 25 °C for 7 days. This process was terminated by adding one drop of Lugol’s solution to each well, and the numbers of unhatched eggs and developed larvae (L_1_–L_3_ stages) in the wells were counted under an inverted microscope. Infective (L_3_) larvae recovered from each microplate well were identified based on both their morphological and morphometric features to the genus level as described by van Wyk et al. [67] and van Wyk and Mayhew [68].

### Questionnaire survey

A questionnaire on putative risk factors for AR development was administered in personal interviews with the person responsible for farm management. This questionnaire (Additional file 1) contained 34 questions divided into four sections: (i) farmer and farming experience, (ii) farm production, farm size, and herd composition, (iii) management practices, and (iv) health issues at the farm (e.g. faecal examination, use of anthelmintics, treatment strategies, alternative approaches to control parasites). The majority (53%) of questions were open-ended (i.e. the respondent had to answer in a few words), approximately a third of the questions were close-ended (i.e. the respondent had to choose from a set of predefined responses), and the remainder (12%) were semi-open questions (i.e. close-ended questions with the additional category “specification”). A copy of the completed questionnaire was sent together with the results of the AR assessment and specific recommendations for sustainable parasite control to the farm owner by e-mail. Any further questions from the farmers were discussed in a telephone conversation.

### Data analysis and assessment of AR and risk factors

A mixed logit model [69] was applied to express the descriptors of toxicological dose for fitting the dose-response EHT and LDT data using nonlinear regression. A medium effective dose (ED_50_), the concentration of TBZ required to prevent 50% of the eggs from hatching, was evaluated in the EHT, and a medium lethal dose (LD_50_), the concentration of IVM-AG required to inhibit 50% of the eggs from developing to the L_3_ stage, was evaluated in the LDT. ED_99_ and LD_99_ were determined concurrently. We followed the recommendations [70, 71] introduced by the COST Action COMBAR (a consortium of scholars from EU countries that focuses on research on AR in helminth parasites of ruminant livestock) when evaluating the results of the EHT; a herd of goats was considered BZ resistant if ED_50_ was > 0.2 μg TBZ.ml^− 1^. The percentage of hatched eggs at 0.3 μg TBZ.ml^− 1^ was also taken into account in evaluating AR based on the study by Babják et al. [12]. Only a low level of resistance was expected in a herd when < 20% of the eggs hatched at 0.3 μg TBZ.ml^− 1^; hatching of 20–50% of the eggs indicated moderate level of AR, and hatching of > 50% of the eggs indicated a high level of AR. An in vitro assessment of IVM resistance requires the definition of discriminating doses for individual nematode species due to the different in vitro sensitivities of the species to IVM. The discriminating dose for in vitro detection of IVM resistance has not yet been clearly established for all species of strongylid nematodes, so we used a concentration of 21.6 ng.ml^− 1^ as a threshold for IVM resistance in this survey, based on previous results [65]. The assessment of the level of IVM resistance was adopted from a survey by Dolinská et al. [72]; a high level of resistance to IVM was expected when > 30% of larvae developed to the infective stage at an IVM-AG concentration of 21.6 ng.ml^− 1^.

The association between farmer characteristics, farm and pasture management, factors of parasite control, and AR status of the herds were evaluated by a univariate logistic regression analysis based on the questionnaire survey. The tests were performed independently for detecting BZ, IVM, and dual resistance (yes/no). The results were expressed as odds ratios (ORs) with the corresponding 95% confidence intervals (CIs). A two-sided Fisher exact test was applied if the model did not converge due to the lack of observations in a particular category. *P* values ≤0.05 were considered significant. The analyses were performed using R statistical software [73].

## Supplementary Information

**Additional file 1.** A questionnaire to gather information about farmer characteristics, farm and pasture management, and parasite control measures. Data on risk factors for anthelmintic resistance development.

## Data Availability

The datasets used and analysed during the current survey are available from the corresponding author on reasonable request.

## Abbreviations

AR: Anthelmintic resistance
BZs: Benzimidazoles
CI: confidence interval
CR: Czech Republic
DMSO: Dimethyl sulphoxide
ED_50_: Medium effective dose
EHT: Egg hatch test
GI: Gastrointestinal
MLs: Macrocyclic lactones
LEV: Levamisole
IVM: Ivermectin
IVM-AG: Ivermectin aglycone
LD_50_: Medium lethal dose
LDT: Larval development test
OR: Odds ratio
SCOPS: Sustainable Control of Parasites in Sheep
TBZ: Thiabendazole
TST: Targeted selected treatment
