# Supplementary material for: Resistance of strongylid nematodes to anthelmintic drugs and driving factors at Czech goat farms
Source: BMC Vet Res. 2021 Mar 5;17:106. doi: 10.1186/s12917-021-02819-8 (PMC7934424; doi:10.1186/s12917-021-02819-8)
Supplement: Supplementary file 1 — Additional file 1. A questionnaire to gather information about farmer characteristics, farm and pasture management, and parasite control measures. Data on risk factors for anthelmintic resistance development. [file 12917_2021_2819_MOESM1_ESM.docx]

**Questionnaire**





Farm identification:

A) **farmer**

1. age:

2. education level:

3. field of education:

4. farming experience:

B) **farm**

5. goat breed:

6. number of goats:

7. commercial production: yes – no, milk production yes – no

8. location (region):

9. altitude:

C) **breeding management**

10. production system: organic – conventional

11. closed herd turnover: yes – no

12. purchasing animals: yes (specify – CR, abroad) – no

13. quarantine for newly purchased animals: yes – no

14. contact with other animals (domestic or wild): yes (specify) – no

15. grazing goats: yes (specify period of grazing) – no

16. nutrition (specify – eg. core feeds, green fodder, feed additives):

17. kidding season (month):

18. weaning (age):

19. milking (specify months):

D) **health issues**

20. parasitological examination: yes (specify – frequency and month) – no

21. anthelmintic drugs:

- drenching frequency:
- month of application:
- anthelmintic class and route of administration (in last 3 years):
- drug dose following the manufacturers’ recommendations (so-called sheep dose) for sheep: yes – no (specify)
- drug administered by a veterinary practitioner: yes – no
- drenching based on parasitological examination: yes – no
- drenching based on clinical symptoms: yes – no
- whole-herd anthelmintic treatment: yes – no
- animals weighed before drug administration: yes – no

22. other (non-chemical) control measures against parasitic infections:

- pasture rotation: yes – no
- bioactive forages supplementation: yes (specify) – no

23. animal health problems (specify):

24. notes (if you have any further information on any point related to the health issues in your herd of goats):
